# Respectful encounters from healthcare professionals and return to work among 9032 long-term sick-listed due to cancer or due to other diagnoses: results from a Swedish population-based survey

**DOI:** 10.1007/s00520-019-4652-4

**Published:** 2019-01-28

**Authors:** Tomas Månsson, Niels Lynøe, Kristina Alexanderson, Elin Hinas, Gert Helgesson, Emilie Friberg

**Affiliations:** 1 Stockholm Centre for Healthcare Ethics, Department of Learning, Informatics, Management and Ethics, Karolinska Institutet, SE-171 77 Stockholm, Sweden; 2 Division of Insurance Medicine, Department of Clinical Neuroscience, Karolinska Institutet, SE-171 77 Stockholm, Sweden

**Keywords:** Cancer, Encounters, Respect, Return to work, Sick leave

## Abstract

**Purpose:**

To examine whether experiences of positive respectful encounters and negative disrespectful encounters differ between sickness absentees with a cancer diagnosis and sickness absentees with other diagnoses, especially in relation to their ability to return to work (RTW).

**Methods:**

A total of 9032 long-term sickness absentees in Sweden responded to a questionnaire (response rate 52%) about experiences of positive and negative encounters with healthcare professionals. The association between different types of such encounters and participants feeling respected or disrespected were calculated with population attributable risk with 95% confidence intervals (CI). The perceived impact on ability to RTW was also examined.

**Results:**

Significantly, larger proportions among those who experienced a positive encounter and also felt respected stated that those encounters facilitated their ability to RTW, compared to those who experienced a positive encounter without feeling respected: among cancer absentees the difference in proportions were 21% (CI, 7–34) versus 50% (CI, 45–55); among absentees with other diagnoses 42% (CI, 37–47) versus 63% (CI, 61–64). Similar comparisons among sick-listed who experienced negative encounters indicated that also feeling disrespected impeded ability to RTW among a significantly larger proportion of those with other diagnoses [51% (CI, 48–54) versus 35% (CI, 31–39) of those not feeling disrespected]. Among cancer absentees, the corresponding proportions were 20% (CI, 9–30) versus 25% (CI, 9–41).

**Conclusions:**

Compared to sickness absentees with other diagnoses, a larger proportion of cancer sickness absentees stated that they were facilitated by respectful encounters and not impeded by disrespectful encounters, regarding self-estimated ability to RTW. More research is needed to examine whether these differences can be associated with use of a patient-centered encountering approach.

## Introduction

Cancer diseases and treatments might influence patients’ health and well-being in different ways. Diagnosis, prognosis, and treatment options are often considered crucial for cancer patients’ possibilities to survive, recover, and—among the now increasing numbers of cancer patients of working ages—return to work (RTW) [1–3]. As for all diagnoses, other factors such as age, sex, educational level, and birth country are of importance for sickness absence duration and RTW [4, 5].

In this context, the quality of the cancer patients’ encounters with healthcare professionals might be considered as of relatively minor importance [6]. Nevertheless, several studies indicate that experiences of respectful encounters with healthcare professionals may result in patients feeling strengthened and encouraged, both among cancer patients and others [7–9], and patients’ experiences of disrespectful or wrongful encounters may have negative consequences for their health and their trust in the healthcare system [9–11]. Moreover, respect for patients and their choices, values, and preferences are often regarded as core elements of patient-centered care [12] and also commonly regarded as a desirable feature of healthcare [12]. Patient-centered care might in turn facilitate shared decision-making [12].

Previous studies have shown that encounters with healthcare staff may influence the self-estimated ability to RTW among long-term sickness absentees [13–19]. These studies indicate that patients’ experiences of positive encounters facilitate their perceived ability to RTW, a tendency that is significantly stronger if the encounter was also perceived as respectful [13–15]. A respectful encounter may strengthen the patients’ self-confidence, which in turn may lead to a greater motivation to overcome difficulties on their way to RTW [14–16].

Correspondingly, negative encounters have been reported to impede the self-estimated ability to RTW, which is accentuated if the patients also felt disrespected [13–15, 20]. It has been suggested that patients’ experiences of disrespectful encounters could be explained by healthcare professionals’ “domination techniques” [11], e.g., not listening to the patient, being contemptuous, and not allowing the patient to question medical expertise [11]. Such experiences of disrespectful encounters may weaken patients’ self-confidence and motivation for RTW [14–16, 20].

Several studies have also shown that an important factor for long-term sickness absentees’ RTW is their experiences of how they were encountered by healthcare professionals [8, 13, 16, 19].

However, so far, there is limited knowledge about whether experiences, perceptions, and consequences of encounters differ between sickness absentees due to cancer and sickness absentees with other sick-leave diagnoses. As a hypothesis, we suggest that patients with cancer diagnoses are not questioned, e.g., regarding their work incapacity, by the healthcare professionals, at least not to the same degree as patients with diagnoses that are more difficult to verify with objective methods. Patients with cancer diagnoses have often been scanned (CT, MR, PET, ultrasound), examined (histopathologically), and tested with diagnostic methods with high accuracy. Similar objective diagnostics might not be available regarding many other patient groups, e.g., patients with low-back pain, neck-shoulder diagnoses, and mental diagnoses.

The large numbers of long-term sickness absentees constitute a great public health problem in many OECD countries [21, 22], and we need more knowledge about what could affect their RTW.

Hence, the aim of this study was to examine whether experiences of positive and respectful encounters and experiences of negative and disrespectful encounters differ between long-term sickness absentees with a cancer diagnosis and sickness absentees with other diagnoses; especially in relation to self-estimated ability to return to work (RTW).

## Methods

In 2013, a comprehensive questionnaire regarding experiences of encounters with healthcare professionals and with officers at the Swedish Social Insurance Agency was sent to a randomly selected sample of 17,395 long-term sickness absentees in Sweden. They were identified by the Social Insurance Agency as having an ongoing sick-leave spell that had lasted for at least 4 and at most, 8 months, corresponding to about half of all people in Sweden with such spells at the time of inclusion. The questionnaire, available only in Swedish, was administrated by Statistics Sweden, who sent questionnaires to the home addresses of the study group, together with information about the study, with three reminders to non-responders. Participants consented by returning the questionnaire. The questionnaire was developed based on previous questionnaires, individual- and group-interviews of long-term sickness absentees, and literature reviews [8, 16, 17, 19, 20].

Statistics Sweden and the Social Insurance Agency provided register data concerning age, sex, educational level, country of birth, and sick-leave diagnosis (categories are presented in Table 1). Regarding the variable self-rated health (obtained from the questionnaire): “How do you rate your general health status?” with the response options: “very good,” “good,” “okay,” “bad,” and “very bad.” We categorized the options “very good” and “good” as “good,” while “very bad” and “bad” were categorized as “bad,” and “okay” as “rather good.” The respondent was also asked to indicate which type of healthcare professional he or she had the most positive/negative encounter with; response options: “physician,” “nurse,” “physiotherapist,” “occupational therapist,” “medical social worker/psychologist,” “naprapath/chiropractor,” “other profession,” and “do not know,”

**Table 1:** Background variables for the participating patients on long-term sickness absence due to cancer and due to other diagnoses

	*Cancer* n (%)	*Other diagnoses* n (%)
Total number	562 (6.4)	8194 (93.6)
Women	377 (67.1)	5715 (69.7)
Age group (years)		
19–34	27 (4.8)	1147 (14.0)
35–44	71 (12.6)	1621 (19.8)
45–54	156 (27.8)	2207 (26.9)
55+	308 (54.8)	3219 (39.3)
Educational level (corresponding years of schooling)		
Elementary school (≤ 9)	59 (10.5)	1034 (12.6)
High school (10–12)	271 (48.2)	4066 (49.6)
University or college (> 12)	230 (40.9)	3075 (37.5)
Self-rated health		
Good	177 (31.4)	2674 (32.6)
Rather good	248 (44.1)	3228 (39.4)
Bad	122 (21.7)	2140 (26.1)
Missing	15 (2.7)	152 (1.8)
Birth country		
Sweden	482 (85.8)	7032 (85.8)

The questionnaire contained 163 questions concerning the participants’ experiences of positive and negative encounters with healthcare professionals and the Social Insurance Agency officers. In this study, we focused on answers regarding the participants’ encounters with healthcare professionals. The questionnaire also asked whether those who had experienced positive encounters also felt respected and whether those who had experienced negative encounters also felt disrespected. The Swedish word “kränkt” was used in the questionnaire and might be translated both in terms of (feeling/being) wronged and disrespected. In the present text, we have used the term “disrespected.”

The participants were asked to answer the question “Did you experience a positive encounter with someone in healthcare in connection to your sick leave?” with the response options: yes/no. Participants who answered yes were asked to specify from a provided list (19 items, of which four were included in the present analysis) what type of positive encounters they had experienced, with four response alternatives for each: “agree completely,” “agree to some extent,” “disagree to some extent,” and “disagree completely,” here dichotomized into agree versus disagree.

Of the many items in the questionnaire, we chose to include responses to the following four types of positive encounters: “listened to me,” “answered my questions,” “believed what I said,” and “provided adequate and correct information.” The choice was partly based on core elements in a patient-centered approach [12, 23, 24], an approach more and more in use in Swedish healthcare.

Correspondingly, the survey included questions about whether they had experienced a negative encounter in connection to their sick leave, and if so what kind of negative encounter (25 items, of which six were included in the present analysis), with the same four response alternatives as stated above. The six items included were identified as being contrary to patient-centeredness: “did not listen,” “interrupted me,” “did not answer questions,” “did not believe what I said,” “doubted my condition,” and “treated me as stupid.”

Finally, the participants were asked whether positive and negative encounters, respectively, had influenced their ability to RTW. For those who had experienced positive (negative) encounters, the response options were: “impeded a lot,” “impeded to some extent,” “had no impact,” “facilitated to some extent,” and “facilitated a lot.” We categorized the options as “impeded,” “no impact,” and “facilitated.”

Response patterns among those with a cancer sick-leave diagnosis were contrasted to the response patterns of sickness absentees with other diagnoses. We divided the two strata into two groups: (1) those who had experienced only positive encounters and (2) those who had experienced only negative encounters and those who had experienced both negative and positive encounters. Participants who had experienced neither negative nor positive encounters were excluded: cancer *n* = 12 (2%), other diagnoses *n* = 264 (3%).

The response rate was 52% and, as often, somewhat higher among women, those of older ages, with higher educational level, and born in Sweden. Among those sick-listed due to cancer, 63% responded.

### Statistics

We calculated population attributable risks (AR) with 95% confidence intervals (CI) [25]. AR was calculated by comparing those who answered that they had experienced only positive encounters and that they also had felt respected (dichotomized into agree/disagree) with specific items, e.g., “listened to me” (agree/disagree). AR takes into account both how common a certain type of encounter is and the strength of the association. If the different types of encounters are entirely independent, the sum proportion of all the ARs would not exceed 100. If ARs total more than 100, we can assume that the experiences of encounters are somehow intertwined. AR was similarly calculated when comparing those who had experienced a negative encounter and also felt disrespected (agree/disagree) with specific items, e.g., “did not listen” (agree/disagree).

When presenting the estimated influence of positive or negative encounters on perceived ability to RTW, we focused on the answers that stated that the encounter had either facilitated or impeded the ability to RTW. These results were presented as proportions with 95% CI.

## Results

**Fig. 1:**
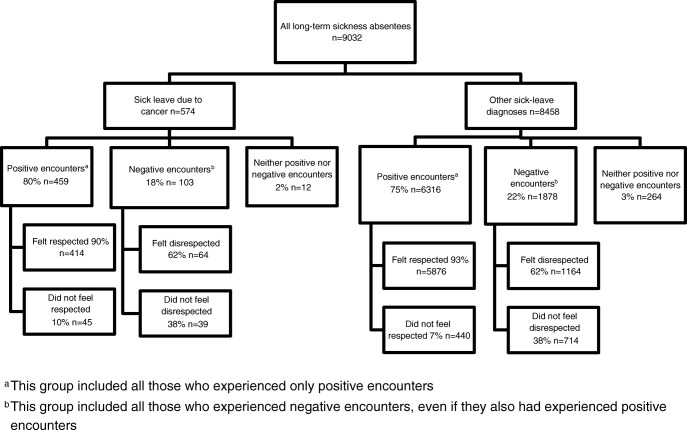
Distribution of long-term sickness absentees due to cancer and other diagnoses, respectively, who participated in the survey, regarding their experiences of positive and negative encounters with healthcare as well as feeling respected and disrespected

Among the 9032 responding participants, 574 (6%) were sick-listed due to cancer and 8458 (94%) due to other diagnoses (Fig. 1). Among sickness absentees with other diagnoses, the most common diagnoses were mental (*n* = 2826), musculoskeletal (*n* = 2734), injuries (*n* = 734), and cardiovascular (*n* = 398) diagnoses.

There were no significant differences between the two groups of patients sick-listed due to a cancer diagnosis or due to another diagnosis regarding background variables such as self-rated health. The proportion of older participants (> 55) were, however, higher in the cancer group compared to the group of patients with other diagnosis (Table 1).

A large majority of the participants, 75%, had only experienced positive encounters (Table 2). Among those with non-cancer diagnoses, i.e.,: cardiovascular diagnoses, 78% had experienced only positive encounters, and, similarly, 76% of those with injuries, 76% of those with a musculoskeletal diagnosis, and 74% of those with a mental diagnosis had experienced only positive encounters. Participants sick-listed with cancer displayed a somewhat higher AR regarding feeling respected and the four specific items analyzed in relation to positive encounters (“listened to me,” “answered my questions,” “believed what I said,” and “provided adequate and clear information”) compared to those with other diagnoses. However, these differences were not significant.

**Table 2:** Distributions of participants who experienced different types of positive encounters among long-term sickness absentees with cancer (*n* = 459) and with other diagnoses (*n* = 6316), respectively, and distributions of participants who experienced different types of negative encounters, among long-term sickness absentees with cancer (*n* = 103) and with other diagnoses (*n* = 1878), respectively. The results are presented as population attributable risks [AR] with 95% confidence intervals (CI) for feeling respected when experiencing different types of positive encounters and for feeling disrespected when experiencing different types of negative encounters

	Cancer	Other diagnoses
	*n* [AR (95% CI)]	*n* [AR (95% CI)]
Type of positive encounter		
Listened to me	433 [79% (58–90)]	5817 [77% (72–82)]
Answered my questions	408 [76% (57–88)]	5758 [65% (61–70)]
Believed what I said	400 [61% (46–74)]	5740 [59% (54–64)]
Provided adequate information^a^	395 [53% (39–66)]	5590 [46% (43–50)]
Type of negative encounter		
Did not listen	44 [30% (12–46)]	911 [37% (32–42)]
Treated me as stupid	35 [18% (4–31)]	834 [37% (33–41)]
Doubted my condition	33 [9% (−5–23)]	932 [35% (30–41)]
Interrupted me	18 [7% (0–15)]	668 [23% (20–26)]
Did not believe what I said	30 [4% (−9–18)]	924 [37% (32–42)]
Did not answer questions	35 [3% (−12–19)]	834 [25% (21–30)]

^a^Provided adequate and clear information/advice

A minority of the participants, 23%, had experienced negative encounters (Table 2). Among those with non-cancer diagnoses, 26% of those with mental diagnoses, 21% of those with musculoskeletal diagnoses, 20% of those with injuries, and 19% of those with cardiovascular diagnoses had experienced a negative encounter. Participants sickness absent with cancer displayed a significantly lower AR regarding feeling disrespected and five of the chosen six items relating to negative encounters (“treated me as stupid,” “doubted my condition,” “interrupted me,” “did not believe what I said,” and “did not answer questions”), compared to those sickness absent with other diagnoses. The item with the highest AR in both groups was “did not listen,” but the difference between the two groups with regard to this item was not significant.

Experiences of positive and respectful encounters were correlated to facilitation of the self-estimated ability to RTW. Comparing those in the two groups (cancer/other diagnoses) who had experienced a positive encounter and also felt respected with those who had experienced a positive encounter without feeling respected, there were significant differences (Table 3). Among those sickness absent due to cancer who had experienced positive encounters but did not feel respected, 21% (CI, 7–34) reported that the positive encounters had facilitated their ability to RTW; in comparison, among those who did feel respected, 50% (CI, 45–55) reported that positive encounters had facilitated their ability to RTW. The corresponding proportions among those with other diagnoses were 42 (37–47) and 62% (61–64), respectively.

**Table 3:** Displays proportions (%) with 95% confidence intervals (CI) among long-term sick-listed patients regarding how experiences of respectful/disrespectful encounters and association with self-estimated ability to return to work (facilitating, not influencing, impeding, respectively), among those long-term sick-listed due to cancer and due to other diagnoses

Return to work was:	Facilitated	Not influenced	Impeded
	% (95% CI)	% (95% CI)	% (95% CI)
Positive encounters			
Sick-listed due to cancer			
Felt respected (*n* = 386)	50.3% (45.3–55.3)	49.2% (44.2–54.2)	0.5% (0.0–1.2)
Did not feel respected (*n* = 34)	20.6% (7.0–34.2)	76.5% (62.2–90.8)	2.9% (0.0–8.5)
Sick-listed with other diagnoses			
Felt respected (*n* = 5684)	62.5% (61.2–63.8)	36.3% (35.1–37.5)	1.2% (0.9–1.5)
Did not feel respected (*n* = 380)	42.1% (37.1–47.1)	56.6% (51.6–61.6)	1.3% (0.2–2.4)
Negative encounters			
Sick-listed due to cancer			
Felt disrespected (*n* = 56)	8.9% (1.4–16.4)	71.4% (59.6–83.2)	19.7% (9.2–30.0)
Did not feel disrespected (*n* = 28)	7.1% (0.0–16.6)	67.9% (50.6–85.0)	25.0% (9.0–41.0)
Sick-listed with other diagnoses			
Felt disrespected (*n* = 1105)	3.8% (2.7–4.9)	45.6% (42.7–46.7)	50.6% (47.7–53.5)
Did not feel disrespected (*n* = 621)	6.0% (4.9–7.9)	58.8% (55.9–62.7)	35.2% (31.5–39.1)

Comparing those in the two groups who experienced negative encounters and also felt disrespected with those who experienced negative encounters without feeling disrespected, there were significant differences among those with other diagnoses, but not among those with cancer, regarding their estimations of whether negative encounters had impeded their ability to RTW (Table 3). Among those sickness absent with cancer who had experienced negative encounters and felt disrespected, 20% (CI, 9–30) reported that their experiences had impeded their ability to RTW; in comparison, among those who did not feel disrespected, 25% (CI, 9–41) reported that their experiences had impeded their ability to RTW. Corresponding proportions among those with other diagnoses were 51 (CI, 48–54) and 35% (CI, 31–39).

## Discussion

Our study indicates that a large majority in both groups of sickness absentees (cancer and other diagnoses) had experienced positive encounters with healthcare professionals, and that they also felt respected. It further indicates that among those who had experienced a positive encounter and also felt respected, a significantly larger proportion stated that those encounters had facilitated their ability to RTW, compared to those who had experienced a positive encounter without feeling respected. Compared to those who were sickness absent due to other diagnoses, a lower proportion of the participants with a cancer diagnosis felt disrespected. Also, a smaller proportion of cancer absentees seemed to be influenced by experiences of negative and disrespectful encounters with regard to their self-estimated ability to RTW compared to those with other sick-leave diagnoses.

Feeling respected was strongly associated with being listened to, getting ones’ questions answered, being believed in, and getting adequate information—items which are core elements in patient-centered care [12, 23, 24]. Regarding feeling disrespected and experiences of items, smaller proportions of participants with a cancer diagnosis, compared to participants with other diagnoses, felt disrespected and answered that the healthcare professional treated them as stupid, interrupted them, disbelieved them, doubted their condition, and did not answer questions.

The present study does not provide any explanations to the identified differences in experiences of encounters between patients sick-listed with cancer and patients with other diagnoses. The differences likely depend on many things, but one explanation might be that the difference in response patterns between the two groups is the result of having encountered different consultation styles.

Very few of the patients with cancer stated that the healthcare professional did not answer questions compared to patients with other diagnoses. This might indicate that such questions are easier to answer or that a larger proportion of the healthcare professionals involved in treating cancer patients apply a patient-centered approach. Being listened to and being believed in are other important aspects of patient-centered care [12, 23, 24]. Very few of the participants with cancer had experienced that healthcare professionals disbelieved them, a significant contrast to those with other diagnoses. A similar difference was also noted regarding the item doubting the patient’s condition. Again, these differences might be explained by the presence or absence of patient-centered care. It should be noted, however, that in Sweden, there are no special formal education goals or requirements regarding communicative competence or patient-centered care for physicians undergoing specialist training in oncology [26] or specialist oncology nurses [27], compared to the formal goals or requirements for other specialist physicians and nurses [26, 27]. Nevertheless, there may be local differences with regard to the training of communication skills. Maybe there are also differences with regard to implemented routines and joint policy concerning handling of work tasks related to sickness certification of patients [28, 29].

The identified differences in experiences of encounters could likely also be explained by the types of examinations cancer patients go through. Cancer patients have most often had their diagnosis verified with diagnostic methods of high accuracy. This provides the healthcare staff with strong reasons to believe in the diagnosis and, perhaps, therefore, motivates them to be more willing to listen to and believe the patients when they describe their symptoms. This stands in contrast to diagnoses where the diagnostic tests are less objectively verifiable, as in the case of low-back pain, neck-shoulder disorders, and common mental disorders; that is, the most prevalent long-term sick-leave diagnoses. Such diagnoses, as well as the level of work incapacity they may lead to, may be more frequently disputed by healthcare professionals due to the kind of diagnostic tools that is available [30]. This might, in turn, influence how patients with these types of symptoms are encountered.

More research is needed in order to reach a well-founded explanation to the differences between patients sick-listed due to cancer and those sick-listed due to other diagnoses regarding their experiences of healthcare encounters.

### Strengths and limitations

The present study is, to our knowledge, the first one comparing long-term sickness absentees’ experiences of encounters with healthcare related to different diagnostic groups. Strengths of the study are the large study population, with people from all over Sweden, and the high number of detailed questions about experienced encounters. The response rate of 52% can be seen as high, considering that many in the study group were very ill and that all had to be able to read Swedish. Nevertheless, 48% did not answer and we do not know how they would have answered. As in all surveys, the questions could have been interpreted in different ways by the participants. Another limitation is that since this was a cross-sectional survey study, we do not know to what extent those who rated their ability to RTW actually did RTW. However, other studies have shown that self-rated ability to RTW is strongly associated with actual RTW [1].

## Conclusion

Long-term sickness absentees with cancer diagnosis were to a larger proportion facilitated by respectful encounters, and less impeded by disrespectful encounters, regarding their self-estimated ability to RTW, compared to long-term sickness absentees with other diagnoses. Moreover, a smaller proportion of the long-term sickness absentees with a cancer diagnosis experienced that they did not get their questions answered, were not listened to, had their condition questioned, or not being believed. This might indicate that their care was more patient-centered, but the difference might also be explained by a higher accuracy in the diagnostic methods used to determine their health status. More research is needed in order to examine possible explanations for these differences.
